# An Anecdote Concerning Dr. Ricord

**Published:** 1890-03

**Authors:** 


An Anecdote Concerning Dr. Ricord.—The following anecdote is related of the great specialist Ricord, lately deceased, and is well worthy of repetition as illustrating the character of that remarkable man.

A poor day laborer, suffering from edema of the glottis, as a result of syphilitic ulceration of the larynx, was brought into the amphitheatre. The unfortunate sufferer was without pulse or respiration, and seemed as good as dead. Prof. Ricord quickly opened the trachea by severing three or four of the tracheal rings, and, overcoming the natural repugnance which anyone would feel in a similar case, applied his lips to the opening and sucked the poisonous matter from the trachea until the patient was again able to breath freely.

Dr. Ricord, with his face covered with blood from the wound, did not even think of washing his face, until the patient was out of immediate danger. It is pleasant to record that the man recovered under appropriate treatment.—*Medical Age*.

